# Rapid cultural adaptations for scalable dissemination of a single‐session intervention among Polish and Ukrainian youth: An open pilot trial

**DOI:** 10.1111/aphw.70099

**Published:** 2026-01-21

**Authors:** Ian Sotomayor, Liliya Morska, Cosette Fox, Piotr Mamcarz, Juan Pablo Zapata, Ewa Domagala‐Zyśk, Dorota Miszczyszyn, Anna Englert‐Bator, Olga Palamarchuk, Andrew Rambadt, Jessica L. Schleider

**Affiliations:** ^1^ Dept of Medical Social Sciences, Feinberg School of Medicine Northwestern University United States; ^2^ Foreign Languages for Sciences Dept Lviv Ivan Franko National University Ukraine; ^3^ Institute of Pedagogy University of Rzeszow Poland; ^4^ Dept of Psychology Holy Cross College United States; ^5^ Dept of Psychology of Emotions & Motivation The John Paul II Catholic University of Lublin Poland; ^6^ Dept of Special Pedagogy The John Paul II Catholic University of Lublin Poland; ^7^ Institute of Psychology University of Rzeszow Poland; ^8^ Psychology & Social Work Dept Vinnytsia Mykhailo Kotsiubynskyi State Pedagogical University Ukraine

**Keywords:** Growth Mindset, Mental Health, Poland, Single‐Session Interventions, Ukraine, Youth

## Abstract

Adolescents across the globe experience increasing demands for care, and the mental health of Polish and Ukrainian youth is especially concerning, due to ongoing war and displacement. This study explores the acceptability, feasibility, and short‐term effects of a digital, self‐guided single‐session intervention (SSI) for improving the mental health of Polish and Ukrainian youth, including Ukrainian refugees in Poland.

A non‐randomized, open pilot trial was conducted from March to June 2024, involving youth aged 10–18 years from Poland and Ukraine. Participants completed an SSI after cultural adaptations and translation into Polish and Ukrainian. Measures assessed hopelessness, self‐hate, agency, perceived control, and acceptability. Statistical analyses included paired t‐tests and effect size calculations to examine intervention effects.

Among 176 Polish and 139 Ukrainian youth who began the intervention, completion rates were 80.7% and 62.6%, respectively. Polish participants exhibited significant improvements in hopelessness, self‐hate, perceived control, and agency, while Ukrainian youth showed moderate improvements in perceived control but limited change in other mental health indicators. Acceptability ratings were high across all youth.

Findings suggest SSIs hold potential as a scalable option for mental health care. However, the varied outcomes across the two groups highlight the need for further refinement, especially for displaced youth.

## INTRODUCTION

Research shows that up to one in five adolescents globally experience mental health challenges (Kieling et al., 2011; Sawyer et al., 2000), with evidence showing these rates are steadily increasing (Bor et al., 2014). Unfortunately, youth can face significant barriers to accessing care, including accessibility issues, provider shortages, and restrictive policies (Benton et al., 2021; O'Brien et al., 2016). These demands for care are further amplified in Central and Eastern Europe, where adolescents are subject to additional sociopolitical and economic pressures (Śliwerski & Kossakowska, 2025). In Poland, the threat of armed conflict with neighboring countries, even in the absence of active warfare, poses both direct and indirect risks to the physical and mental health of youth (Wójtowicz‐Szefler et al., 2023). For Ukrainian adolescents, including both internally displaced and non‐displaced youth, recent studies have begun to document the mental health impacts of the Russo‐Ukrainian conflict, including heightened levels of anxiety, stress, and depression (Fornaro et al., 2025; Palace et al., 2024). Unfortunately, global mental health care systems have historically been ill‐prepared to address these emerging challenges, particularly in the context of armed conflict and war‐like situations (Mollica et al., 2004). Such circumstances often create critical barriers to accessibility, including infrastructure destruction, supply shortages, and the displacement of mental health care workers (Islam & Mozumder, 2021). These disruptions further strain healthcare systems, making it increasingly difficult for both providers and affected populations to access essential health care (Seyedin et al., 2021). Consequently, there is an urgent need for adaptable mental health care services capable of addressing these rapidly evolving conditions, especially as rates of mental health distress continue to escalate among youth.

### Single‐Session Interventions

A systematic review on the mental health challenges faced by youth affected by the Russo‐Ukrainian conflict reveals that current interventions remain insufficient (Fornaro et al., 2025). Efforts thus far have largely relied on traditional, in‐person psychological treatments, which are challenging to widely implement, especially in crisis‐affected regions. While there has been a rise in alternative approaches, such as art, music, and drama therapies, aimed at supporting youth mental health (Fornaro et al., 2025), these interventions demonstrate limited clinical impact. Studies evaluating their effectiveness frequently report minimal improvements in mental health symptoms (Gever et al., 2023; Kim et al., 2023). Although these therapies may offer therapeutic value, youth exposed to armed conflict often face compounded traumatic experiences, including the loss of loved ones, displacement, and the fragmentation of their communities. Addressing the mechanisms through which such trauma leads to adverse psychological outcomes is critical (Bosqui & Marshoud, 2018).

One promising approach to meet these challenges is the implementation of digital, self‐guided single‐session interventions (SSIs). SSIs are brief, self‐administered programs that incorporate core principles of evidence‐based treatments, making them highly scalable across diverse care settings (Schleider et al., 2020). In contrast to traditional therapy, which typically involves multiple sessions over weeks or months, SSIs deliver therapeutic content in a single, often self‐guided session (Schleider et al., 2019; Schleider & Weisz, 2018). A growing body of evidence demonstrates that SSIs result in significant, sustained improvements in mental health outcomes, including reductions in depression, anxiety, hopelessness, and suicidal thoughts and behaviors. An umbrella review synthesizing data from 24 systematic reviews, covering 415 SSIs, found positive outcomes in 83.30% of the reviews analyzed (Schleider et al., 2025). This evidence highlights the potential of SSIs to address the rising mental health needs of youth affected by the Russo‐Ukrainian War. However, to date, no SSIs have been specifically developed for this population.

### Growth Mindset Single‐Session Intervention

While adverse traumatic events are commonly associated with harmful psychological consequences, recent trauma research highlights that such experiences can also act as catalysts for growth (Cryder et al., 2006; Zoellner & Maercker, 2006). This perspective underscores the potential to foster the belief that, while challenging, trauma can lead to positive changes, such as increased resilience and a deeper appreciation for life (Forgeard, 2013; Rhee et al., 2013; Seidmahmoodi et al., 2011; Taku et al., 2015). Adolescence, in particular, represents a critical developmental stage for fostering these transformations. Youth are thought to adapt to challenges more readily than adults due to factors such as heightened brain plasticity, less rigid expectations, and a greater openness to learning and exploration (Fivush et al., 2011; Hafstad et al., 2010; Hamond & Fivush, 1991). Given these considerations, a promising approach for an SSI aimed at supporting the mental health of Polish and Ukrainian youth involves promoting a growth mindset. By empowering youth to transform the effects of their traumatic experiences, the harmful consequences of war and displacement might be mitigated.

Growth mindset interventions hold particular promise for war‐affected youth because of their potential to catalyze personal growth and resilience to help youth better cope with the psychological impacts of war and displacement (Burnette et al., 2022; Shah & Mishra, 2021). Adolescence is a period of remarkable neurodevelopmental plasticity, during which experiences can profoundly reshape neural circuits (Ellwood‐Lowe et al., 2022). Even following traumatic disruption, the adolescent brain remains capable of forming new connections. By nurturing beliefs about personal growth, a growth mindset intervention promotes the rewiring of pathways compromised by stress, thereby restoring a sense of self‐efficacy. Trauma undermines an individual's belief in their own agency, leading to learned helplessness, but by reframing setbacks as opportunities for development (i.e., “mistakes are just information,” as described by Masten & Narayan, 2012), the intervention rebuilds experiences of mastery that are essential for regaining confidence. Importantly, we recognize the limitations of a purely cognitive approach when adversity is ongoing. In contexts of active conflict or repeated displacement, continuous threats often overwhelm simple reframing strategies (Tol et al., 2023). This recognition guided our cultural adaptations to ensure the intervention balanced growth mindset principles with trauma‐sensitive content appropriate for youth experiencing ongoing stressors.

One evidence‐based SSI that fosters a growth mindset is Project Personality, which has been tested with thousands of adolescents in the United States (Schleider et al., 2022) and other contexts (e.g., Buchlmayer et al., 2024). The intervention has demonstrated short‐ and long‐term mental health benefits, including reductions in symptoms of depression and anxiety (Schleider et al., 2019, 2022). While Project Personality was designed to appeal broadly to diverse youth, research indicates that the effectiveness of growth mindset interventions may vary across contexts and populations (Burnette et al., 2022). Context plays a pivotal role in shaping clinical and implementation outcomes, as fostering a growth mindset relies on understanding the social and cultural nuances associated with these beliefs (Burnette et al., 2022). Furthermore, inadequate cultural adaptation of growth mindset or mental health interventions poses significant risks. Without sufficient tailoring, interventions may lead to reduced adherence, unintended harmful practices, and increased distrust in mental health services (Cabassa & Baumann, 2013). This highlights the importance of culturally informed approaches to ensure positive outcomes and mitigate potential harm.

### Present Study

Despite the critical importance of cultural adaptation, existing efforts to adapt mental health interventions are often time‐consuming, costly, and logistically demanding. In conflict‐affected settings, where vulnerable populations face urgent mental health needs, demands for care may clash with limited funding, insufficient human resources, poor security conditions, and logistical challenges that further hinder the ability to deliver culturally adapted interventions (Ager et al., 2019). Addressing these barriers is essential to ensure effective and ethical delivery of mental health care in these contexts.

This study seeks to address this gap by presenting a protocol for the rapid cultural adaptation of a brief, evidence‐based growth mindset SSI (i.e., Project Personality). By doing so, it aims to contribute to the growing body of knowledge on practical, scalable approaches to cultural adaptation, ensuring mental health interventions are both effective and contextually appropriate for vulnerable populations. Specifically, this study aims to answer the following research questions: (1) To what extent is the adapted SSI acceptable and feasible for Polish and Ukrainian youth? (2) Is completion of the SSI associated with short‐term improvements in key mental health outcomes? (3) How do the unique sociocultural and war‐related circumstances of Polish and Ukrainian youth, particularly refugees, influence their engagement with and outcomes from a brief digital psychotherapeutic intervention?

For the current study, Project Personality was adapted and piloted among youth recruited through their schools in Poland. The intervention was made freely available on an online platform (https://www.schleiderlab.org/yes.html). After linguistic and cultural adaptations (described below), we evaluated youth ratings of Project Personality's acceptability and its immediate effects on proximal, clinically relevant outcomes, including hopelessness, agency, self‐hate, and perceived control. Immediate effects on positive mental health were further assessed in a subgroup of Polish and Ukrainian youth. Additionally, we examined usage patterns, including completion rates and duration of use, as well as demographic variables such as age range and sex assigned at birth, with certain demographic questions tailored specifically to Polish or Ukrainian youth. Acceptability metrics assessed whether adolescents perceived the digital, self‐guided SSI as valuable, helpful, and user‐friendly. As this project was a program evaluation of an open pilot trial, the study was largely exploratory with two primary hypotheses. First, we hypothesized that Polish and Ukrainian youth who completed the SSI would report significant decreases in hopelessness and self‐hate, alongside significant increases in agency and perceived control, from pre‐ to post‐intervention. Second, based on prior research suggesting culturally adapted interventions are rated favorably (Buchlmayer et al., 2024), we hypothesized that participants would rate the SSI as acceptable, with a mean score of three or greater on a five‐point scale.

## METHODS

### Sample Characteristics and Recruitment

Participants between the ages of 10 and 18 were recruited from Poland and Ukraine between March 2024 and June 2024 using opportunistic sampling (i.e., if a school agreed to permit researchers to visit and conduct the study). This study was designed and conducted as a non‐randomized, anonymous program evaluation and prior to initiating the study, all procedures were reviewed and approved by the Holy Cross College Institutional Review Board (IRB) in the US state of Indiana. In Poland, Polish students were recruited from two schools in the city of Rzeszow and one school in the city of Lublin by randomly selecting classes to visit, where the entire class was directed to visit a website (e.g., https://www.schleiderlab.org/yes.html) and complete the Polish‐translated SSI. Students were not informed about the purpose of the visit beyond the information included on consent forms for their parents to sign. Faculty from the Department of Education and the Psychology Institute at the University of Rzeszow and faculty from the Department of Emotion and Motivation Psychology at John Paul II University of Lublin were the study team representatives who contacted Polish schools and recruited Polish youth between ages 10 to 17 years old. These faculty contacted each school's principals and received approval to run the study in randomly selected classrooms. Each school assigned a point person to collect parental consent and coordinate the details of the sessions guiding youth to the online SSI for completion, as approved by the IRB. Parents received consent forms in Polish describing the gist of the study and providing IRB contact information in case of questions or concerns. Consenting parents were asked to fill in the form, sign it, date it, and return it to school. Nonconsenting parents were instructed to write their name with a “No” marked next to it and to also return the form to school. On the day youth anonymously completed the online activity, verbal assent was received from willing participants, and the research team from the United States gave a brief description of the study to the participants. Students who provided assent participated in the online intervention translated into Polish using their mobile phones. Some students had turned 18 years old during the academic school year and returned the parental consent forms with their own signatures, indicating their consent. Following the intervention, participants were encouraged by the researchers to ask questions and further discuss the concept of growth mindset and its association with mental health.

Ukrainian youth between ages 10 and 17 years old were recruited from both Poland and Ukraine using opportunistic sampling (i.e., all available students in the refugee center and Ukrainian schools were invited to participate). In Poland, the research team was able to recruit Ukrainian participants through a collaboration with a Ukrainian Refugee Center in Lublin run by Caritas, the largest humanitarian organization in Poland. Before the arrival of the research team, the Ukrainian Refugee Center contacted the Ukrainian families they serve in the region and collected parental consent forms just like described earlier except the forms were translated into Ukrainian. On the day of anonymous program evaluation, Ukrainian youth were gathered in one large room and the research team repeated the same procedure described above. Participants had a choice of doing the online intervention in Polish or Ukrainian using their mobile phones or tablets provided during the session. Participants under 18 years of age provided their assent before beginning, and those who were 18 or older signed the parent consent forms themselves. Researchers followed the same procedure post‐intervention described above.

In Ukraine, faculty from the Psychology Department at Vinnytsia State Pedagogical University reached out to the Educational Government Unit of Vinnytsia to recruit students as participants in the research study. A government representative from that unit assigned the researchers to a school where the study would be conducted. The school administration collected parental consent as described above and gathered students from various grades ranging between the ages of 10–17 years old. The Ukrainian collaborators followed the same procedures described above and guided the participants using their mobile phones or tablets provided during the session to the Ukrainian translation of the intervention.

### Design

This non‐randomized, anonymous, and open pilot study examined the effectiveness and acceptability of a growth mindset SSI (i.e., named “Project Personality”) among Polish and Ukrainian youth. In order to detect significant changes in pre‐ to post‐SSI measures, approximately 30 complete participant responses (n = 30) are needed to effectively power the analyses, as established in previous trials (see osf.io/e52p3 and Schleider et al., 2020) using G*Power to determine 80% power at α =. 05 for large effects (Schleider et al., 2020). Therefore, for Polish youth, Ukrainian youth in Ukraine, and Ukrainian refugee youth in Poland, analyses were performed after a minimum of 30 participants were recruited for each group.

### Cultural Adaptation Procedures

Project Personality (Schleider & Weisz, 2018, 2019) was systematically translated and culturally adapted into Polish and Ukrainian following established guidelines for the cross‐cultural adaptation of psychological instruments (Borsa et al., 2012; Cruchinho et al., 2024) and the rapid adaptation of interventions in humanitarian settings (Perera et al., 2020). The goal of this study, as with all cultural adaptations, was to determine the changes necessary to make the intervention protocols more meaningful and acceptable to beneficiaries (Griner & Smith, 2006). The adaptation process revealed that effective cultural translation required more than linguistic accuracy; it demanded trauma‐informed modifications that acknowledged the ongoing nature of war‐related stressors. Adaptations ensured the intervention remained both therapeutically sound and culturally resonant for Polish and Ukrainian youth experiencing varying degrees of war‐related stress. Examples of key cultural adaptations are displayed in Table 1.

**TABLE 1:** Examples of key cultural adaptations for Polish and Ukrainian versions.

Original content	Cultural/Trauma issue	Adaptation	Rationale
**Phineas Gage story with “railroad spike” imagery**	Triggering violent imagery for war‐affected youth	**Ukrainian**: Modified with “milder” language, removed graphic images **Polish**: Retained story but added contextual warnings	Ukrainian professionals noted teens linked imagery to bomb injuries; required sensitive rewording to avoid triggering youth who experienced explosions or lost loved ones in combat
**Brain and neuron diagrams (pages 6–7)**	Too complex for younger participants	**Both versions**: Simplified to popular‐science format with age‐appropriate imagery	Adapted to match school curriculum knowledge; ensured 12–13 year‐olds could understand concepts
**Self‐hate items**: “I hate myself,” “I feel disgusted when I think about myself”	Harsh language problematic for vulnerable youth	**Ukrainian**: “Do you sometimes feel disappointed in yourself?” “There are times when I feel I've let myself down” **Polish**: Retained original with contextual framing	Ukrainian youth in focus groups found original wording “too harsh”; vulnerability heightened by war‐related stress required gentler phrasing
**Neuroplasticity metaphor**: “Brain connections like pencil marks”	Preference for agency‐based over biological explanations	**Polish**: “Możesz zmienić swoje myślenie i zachowanie poprzez własne decyzje” (You can change through your own decisions) **Ukrainian**: Emphasized resilience and personal strength	Cultural values emphasize personal agency and spiritual growth over biological determinism
**Example**: “Starting at a new school”	Limited relevance to refugee experiences	**Ukrainian**: “When Maria had to leave her home and start school in Poland, not knowing the language...” **Polish**: Added economic migration context	Reflects displacement experiences, language barriers, and forced adaptation
**Crisis resources**: US helplines (Crisis Text Line, 1–800‐273‐TALK)	Irrelevant for target population	**Both versions**: Replaced with domestic mental health resources and local crisis lines	Ensured youth had access to culturally appropriate, accessible support services
**“People do not stay sad forever”**	Oversimplification given ongoing trauma	**Both versions**: “Trudne uczucia mogą się zmieniać z czasem, choć czasem potrzeba wsparcia” (Difficult feelings can change over time, though sometimes support is needed)	Acknowledges complexity of trauma recovery while maintaining hope

The adaptation process for the Polish and Ukrainian versions followed identical methodological procedures but was conducted independently by separate teams. Each team initially recruited three bilingual experts proficient in both the source and target languages, familiar with psychological terminology, and knowledgeable about youth mental health concepts. These experts independently created three forward translations for each language, focusing on conceptual rather than literal equivalence, with an emphasis on age‐appropriate vocabulary and culturally relevant examples. The forward translations were synthesized through collaborative meetings involving the principal investigators and translators. During these meetings, each translation was reviewed item by item to identify discrepancies in terminology, cultural references, and idiomatic expressions. This process produced preliminary unified translations for each language that preserved the psychological constructs of the original intervention while ensuring linguistic and cultural appropriateness. Particular challenges arose in translating growth mindset terminology, which required meticulous efforts to maintain conceptual integrity while using natural, culturally relevant expressions in the target languages. For the back‐translation phase, three additional bilingual experts for each language, who had no prior exposure to the original intervention, independently translated the synthesized versions back into English. These back‐translators were selected based on their proficiency in English as their primary language and their strong competence in the target language. The back‐translations were then compared to the original English version to identify conceptual errors, mistranslations, or semantic shifts.

The final linguistic adaptation involved members of the investigative team and an eight‐member multiprofessional committee, including psychologists, pedagogists, linguists, and cultural consultants. This committee conducted systematic comparisons of the original, synthesized, and back‐translated versions. The analysis focused on semantic equivalence (ensuring words conveyed the same meaning), idiomatic equivalence (adapting colloquialisms appropriately), experiential equivalence (ensuring scenarios were culturally relevant), and conceptual equivalence (preserving theoretical constructs across cultures). As linguistic adaptations progressed, team members identified components of the intervention that could be culturally adapted using the eight dimensions of the Ecological Validity Model (Bernal et al., 1995). Potential sources of cultural non‐fit, such as somatic expressions of distress and taboo topics, were also noted. These potential issues were refined into specific questions for focus group discussions with mental health professionals specializing in youth psychology (n = 6 for Polish; n = 5 for Ukrainian). All professionals were native speakers of the target languages. They assessed the intervention's cultural appropriateness, identifying problematic expressions or examples. In the Polish version, several metaphors were adjusted to align with cultural norms, while the Ukrainian version required modifications to integrate post‐conflict resilience narratives relevant to the target population.

Before the program evaluation, focus group interviews were conducted with samples from the target populations, consisting of students aged 13 to 17 (n = 6 for each language). These participants completed the intervention in controlled settings, during which researchers documented comprehension challenges, emotional reactions, and engagement levels. Semi‐structured interviews followed, assessing the content's clarity, cultural relevance, and acceptability. This process highlighted instances where terminology, while technically accurate, was unfamiliar to youth, prompting vocabulary adjustments. Additionally, testimonial examples deemed culturally incongruent were replaced with scenarios reflective of Polish and Ukrainian adolescents' lived experiences. Final revisions integrated feedback from both expert reviewers and the youth participants. For the Polish version, 14 modifications were made, primarily involving vocabulary simplification and cultural contextualization of examples. The Ukrainian version required 17 modifications, including adjustments for differences in the educational system and the inclusion of culturally resonant metaphors. All changes were documented and reviewed by the original developers to ensure fidelity to the intervention's core mechanisms.

The finalized Polish and Ukrainian versions were implemented on the Project YES platform (https://schleiderlab.org/yes.html), with language options prominently featured on the English‐language interface. Due to platform constraints, pre–post measures, including the Children's Revised Impact of Event Scale (CRIES‐8) and the Psychological Mindset Health Scale (PMHS), were administered only to the first wave of participants (n = 101 for Polish; n = 28 for Ukrainian) to collect preliminary validation data. Both versions underwent technical testing to confirm functionality across various devices and browsers before being made freely accessible for wider dissemination and subsequent effectiveness evaluations.

### Intervention

The SSI, titled “Project Personality” (Schleider & Weisz, 2018, 2019), has been translated into at least seven languages, some of which have been tested empirically, such as in Spanish (Shroff et al., 2023) and Arabic (Buchlmayer et al., 2024). The 30‐minute, self‐guided youth program includes the following: (1) an introduction to the brain and a lesson on neuroplasticity; (2) testimonials from older youths who describe their views that traits such as personality are malleable, due to the brain's plasticity; (3) further stories by older youths, describing times when they used “growth mindsets” to persevere during social/emotional setbacks; (4) study summaries noting how/why personality can change; and (5) an exercise in which youths write notes to younger students, using scientific information to explain people's capacity for change. The SSI was originally designed and tested with adolescents (Schleider & Weisz, 2016). Materials for the English translation of the SSI are publicly available via the Open Science Framework (Project Personality, Schleider & Weisz, 2019; https://osf.io/4ydhx).

### Measures

Participant outcomes following Project Personality were assessed using the following measures, administered before and after the SSI:

#### Demographics

Prior to completing the SSI, participants were asked to report demographic information (i.e., age, in ranges of 2 years to maintain anonymity) in both translations, with specific demographic information depending on Polish or Ukrainian translation.

Polish demographic questions included nationality (i.e., Polish, Ukrainian, German, Czech, Slovakian, Belarusian, Lithuanian, Russian, other), sex assigned at birth (i.e., male, female, other; translated as “what is your gender?”), time spent in Poland (if “Polish” is not selected for nationality), and refugee status (i.e., yes, no, other).

Ukrainian demographic questions included sex assigned at birth (i.e., male, female, other; translated as “enter your gender”), arrival in Poland (i.e., “before the start of military events in 2014,” “after 2014 but before the Russian invasion in 2022,” “after the full‐scale invasion by on February 24th, 2022”), refugee status (i.e., yes, no), living arrangements prior to Poland, and specifying which family members are currently living with the youth.

#### Mood and Feelings Questionnaire: Short Version

The Mood and Feelings Questionnaire, Short version (SMFQ) is a valid, reliable screening tool for depression in adolescents (Lundervold et al., 2016). Prior to completing the SSI, participants rated 13 statements (e.g., “I felt lonely,” “I felt miserable or unhappy”) reflecting thoughts and feelings in the past two weeks on a 3‐point Likert scale (i.e., 0 = “Not true,” 2 = “True”), with total sum scores ranging from 0 to 26. Internal consistency ranged from α = .85 to .91 for Ukrainian and Polish youth. Note that the SMFQ does not measure suicidal ideation or suicidality, which were not assessed in the context of this pilot trial.

#### Children's Revised Impact of Event Scale: 8‐Item Version (CRIES‐8)

The Children's Revised Impact of Event Scale 8‐item (CRIES‐8) is a validated and reliable tool for assessing youths' responses to traumatic experiences (Kankaanpää et al., 2024), with translations in Polish, Ukrainian, and other languages. Before completing the SSI, adolescents rated their agreement with eight statements screening for trauma‐related symptoms by responding with either “Not at all” (= 0), “Rarely” (= 1), “Sometimes” (= 3), or “Often” (= 5). Total score ranges from 0 to 40, with higher scores indicating higher trauma‐related symptoms. Internal consistency ranged from α = .67 to .80 for Ukrainian and Polish youth. Though the Cronbach's alpha was relatively low for the CRIES‐8 administered among Ukrainian youth, the measure was retained because of the strong consistency observed among all other measures.

#### Beck Hopelessness Scale: 4‐Item Version (BHS‐4)

The Beck Hopelessness Scale 4‐item (BHS‐4) is a reliable and shortened version of the 20‐item scale used to measure hopelessness in youth (Forintos et al., 2013). Before and immediately after the SSI, participants rated four statements indicating their current sense of hopelessness (i.e., “right now, in this moment”) on a 4‐point Likert scale ranging from 0 (i.e., Absolutely Disagree) to 3 (i.e., Absolutely Agree). Total score ranges from 0 to 12, with higher scores indicating greater levels of hopelessness. Internal consistency ranged from α = .74 to .83 for Polish and Ukrainian youth across both timepoints (i.e., before and after the SSI).

#### State Hope Scale: Agency Subscale

The State Hope Scale is a 6‐item self‐report scale created to evaluate hope in youths. The State Hope scale includes two reliable subscales: agency and pathways (Snyder et al., 1996). The “agency” subscale measures perceived ability to generate plans and work towards achieving goals (e.g., “I can think of many ways to reach my current goals”) and the “pathways” subscale measures perceived success in meeting those goals (e.g., “At this time, I am meeting the goals I have set for myself”). In this program evaluation of an SSI, we expected shifts in *agency* but not in *pathways* scores, because participants would not yet have had opportunities to pursue or enact goals immediately following SSI completion. Therefore, we indexed hope using the 3‐item agency subscale of the State Hope Scale. Before and immediately after the SSI, participants rated three statements based on how they think about themselves right now on an 8‐point Likert scale ranging from 1 (i.e., Definitely False) to 8 (i.e., Definitely True). Total score ranges from 6 to 48, with higher scores reflecting greater overall agency thinking. Internal consistency for Polish and Ukrainian youth, across timepoints, ranged from α = .70 to .88.

#### Self‐Hate Scale

The Self‐Hate Scale (Turnell et al., 2019) is a reliable, 7‐item measure for assessing feelings of self‐hatred and a shortened, 3‐item version was previously adapted for SSI studies (Schleider et al., 2022). Before and immediately after the SSI, participants rated how true each of three statements was for them in that moment (i.e., “I hate myself,” “I feel disgusted when I think about myself,” “I feel ashamed of myself) on a 6‐point Likert scale (1 = “Not at all true for me; 6 = “Very true for me”). Higher scores indicate greater levels of self‐hate, with possible scores ranging from 7 to 49. Internal consistency ranged from α = .95 to .99 for Ukrainian and Polish youth across both timepoints.

#### Perceived Control

A single‐item measure of adolescent perceived control asked youth to rate their agreement with the statement, “right now, I feel like things are out of my control” on an 11‐point scale ranging from 0 (“Not at all”) to 10 (“A lot”). Pre–post‐SSI reductions in response scores signify improvement, or in other words, a low score post‐SSI indicates the participant feels “more in control” after completing the SSI.

#### Positive Mental Health Scale

The Positive Mental Health Scale (PMHS) is a measure assessing positive mental health as evident in general emotional, psychological, and social well‐being, previously validated among participants from Poland (Velten et al., 2022). Before and immediately after the SSI, participants rated their agreement with nine statements assessing current positive beliefs (e.g., “I am in good physical and emotional condition”) on a 4‐point scale ranging from 0 (i.e., “Do not agree”) to 3 (i.e., “Agree”). Total scores range from 0 to 27, with higher scores representing greater states of positive mental health (e.g., more optimism, joy, calm, etc.). Internal consistency ranged from α = .82 to .91 for Ukrainian and Polish youth across both timepoints.

#### Perceived Change in Hopelessness and Problem‐Solving

Both Polish and Ukrainian youth were asked two questions immediately after completing the SSI, which evaluated any subjectively perceived change in hopelessness and the ability to solve problems. The questions have been replicated from previous SSI trials (e.g., Buchlmayer et al., 2024; Shroff et al., 2023) and were originally based on established guidelines for assessing subjectively perceived change following an intervention (Anvari & Lakens, 2021). These questions asked, “to what extent are you feeling hopeless right now?” and “to what extent are you able to solve the problems you are facing right now?” and both in the context of “compared to before doing this activity.” Both questions prompted participants to rate their response on a 5‐point Likert scale (i.e., “much more hopeless” to “a lot less hopeless; “much less able to solve problems” to “a lot more able to solve problems”). These measures were created based on previously established methods for calculating the “smallest effect size of interest,” or in other words, the smallest possible effect size associated with a detectable, subjective change within individuals (Schleider et al., 2019).

#### Program Feedback Scale

Acceptability was assessed using the Program Feedback Scale (PFS), a scale frequently used to evaluate acceptability and user perceptions of SSIs (Buchlmayer et al., 2024; Schleider et al., 2020; Shroff et al., 2023). The PFS asks participants to rate their level of agreement with seven statements that indicate acceptability and feasibility of the SSI (e.g., “I enjoyed the program”) on a 5‐point Likert scale ranging from 1 (i.e., Really Disagree) to 5 (i.e., Totally Agree). The PFS was adapted from existing, validated acceptability assessments of digital interventions. To exclude items that did not apply to web‐based SSIs (i.e., items that reference one's interest in revisiting the program), adaptations from existing scales were needed. The PFS also assessed participants' open‐response feedback. The PFS item scores can be evaluated either individually or across items via a mean score. Internal consistency across items for youth who completed the SSI in Polish was α = .85, and internal consistency across items for youth who completed the SSI in Ukrainian was α = .91. However, mean responses to *each* individual PFS item were evaluated separately to inform understandings of acceptability in specific domains (e.g., ease of use, enjoyability, ease of understanding).

### Analytic Plan

To analyze usage patterns of the Polish and Ukrainian translations of Project Personality, we quantified the number of youths who completed baseline questionnaires, the 30‐minute self‐guided SSI, questions on acceptability, and post‐SSI questionnaires. We also calculated the average number of minutes spent completing baseline measures, the SSI, and post‐SSI measures. Accordingly, we report dropout rates at various stages of the trial.

Using a sub‐sample of participants who completed the SSI and pre‐ to post‐SSI measures, we reported mean scores and standard deviations on pre‐ and post‐SSI scales assessing hopelessness, agency, self‐hate, perceived control, and positive well‐being. Additionally, we calculated within‐group effect sizes (Cohen's dz including 95% confidence intervals) to report the magnitude of change in pre‐ to post‐SSI levels of each outcome (i.e., hopelessness, agency, self‐hate, perceived control, positive well‐being). Mean ratings above 0 on any change‐related item indicate an overall, subjectively detectable pre‐ to post‐SSI change on that particular measure.

Using a sub‐sample of participants who completed the PFS, we reported means and standard deviations for each item and for the overall score. A mean score above 3 on any given PFS item reflects endorsement of that item (i.e., a positive feedback/adequate acceptability); a mean overall score of >3 (mean of all items) reflects overall perceived SSI acceptability.

We ran a logistic regression to determine whether pre‐SSI depression or trauma‐related symptom severity, or any demographic factor (age range, sex, nationality, refugee status), was significantly associated with (a) survey completion (85% or more – binary variable); (b) SSI completion (binary); (c) change in pre‐ to post‐SSI change measures; and/or (d) mean PFS score. Participants with incomplete data will be excluded on a per‐analysis basis; total sample sizes were reported separately for each test conducted.

#### Data Quality and Exclusions

Research involving self‐administered measures, especially within community‐based program evaluations such as the present study, can often collect data from participants who do not appropriately complete the questionnaires, either because of insufficient attention or intentionally invalid responses. Prior research has demonstrated that even a small percentage of invalid responses undermines the interpretability of results (Arias et al., 2020), therefore it is imperative to identify and filter careless or invalid effort (CIE) responses to optimize data interpretation and refine the accuracy of results. For the current study, specificity was prioritized over sensitivity, meaning the filtering strategy prioritized identifying and discounting only the most clearly CIE responses, rather than filtering all potentially CIE responses. Specifically, data were filtered if individual participant responses exhibited a “straightlining” pattern, whereby a participant provided identical responses to all items of a measure regardless of the content and direction (DeSimone et al., 2018).

## RESULTS

Pre‐registration of this study, anonymized data, and code for all analyses are available on the Open Science Framework using this link: https://osf.io/9h84t/.

### Sample and Usage Patterns in YES

From March 2024 to June 2024, a total of 176 Polish youth and 139 Ukrainian youth began a single‐session intervention (i.e., Project Personality), of whom 142 Polish (80.68%) and 87 Ukrainian (62.59%) youth completed the 30‐minute self‐guided intervention. Polish youth included youth recruited in Poland who accessed the Polish translation of the SSI. Ukrainian youth included youth recruited in both Poland and Ukraine, all of whom accessed the Ukrainian translation of the SSI.

Among Polish youth who were eligible and consented to research participation (N = 160), a majority identified as a girl (70.60%) and were between the ages of 16–17 (63.10%). Nearly all identified their nationality as being Polish (95.00%), although additional nationalities were reported (see Table 2). Only one participant who completed the Polish translation indicated a refugee status (0.63%).

**TABLE 2:** Demographic Characteristics of All Youth.

Demographic Characteristic	Polish Youth; *N* = 160	All Ukrainian Youth; *N* = 107	Ukrainian Youth in Poland; *N* = 25	Ukrainian Youth in Ukraine; *N* = 82
	*n* (%)	*n* (%)	*n* (%)	*n* (%)
Age				
10 or younger	2 (1.25%)	3 (2.80%)	2 (8%)	1 (1.22%)
11 to 12	0 (0%)	24 (22.40%)	2 (8%)	22 (26.80%)
13 to 15	44 (27.50%)	57 (53.30%)	17 (68%)	40 (48.80%)
16 to 17	101 (63.10%)	19 (17.80%)	2 (8%)	17 (20.70%)
18 or older	13 (8.12%)	4 (3.74%)	2 (8%)	2 (2.44%)
Nationality				
Polish	152 (95.00%)	N/A	N/A	N/A
Ukrainian	1 (0.63%)	N/A	N/A	N/A
German	1 (0.63%)	N/A	N/A	N/A
Czech	1 (0.63%)	N/A	N/A	N/A
Belarusian	1 (0.63%)	N/A	N/A	N/A
Other	6 (3.75%)	N/A	N/A	N/A
No Response	6 (3.75%)	N/A	N/A	N/A
Gender				
Boy	39 (24.40%)	36 (33.60%)	4 (16%)	32 (39.00%)
Girl	113 (70.60%)	63 (58.90%)	21 (85%)	42 (51.20%)
Other	2 (1.25%)	3 (2.80%)	0 (0%)	3 (3.66%)
No Response	6 (3.75%)	5 (4.67%)	0 (0%)	5 (6.10%)
Refugee Status				
Yes (refugee)	1 (0.63%)	24 (22.40%)	19 (76%)	5 (6.10%)
No	152 (95.00%)	46 (43.00%)	5 (20%)	41 (50.00%)
No Response	7 (4.38%)	37 (34.60%)	1 (4%)	36 (43.90%)
Poland Arrival				
“Before military events in 2014”	N/A	5 (4.67%)	0 (0%)	5 (6.10%)
“After 2014, before 2022”	N/A	8 (7.48%)	4 (16%)	4 (4.88%)
“After invasion on 2/24/2022”	N/A	45 (42.10%)	21 (84%)	24 (29.30%)
No Response	N/A	49 (45.80%)	0 (0%)	49 (59.80%)
Ukraine Area				
“In occupied territory in eastern or southern Ukraine”	N/A	4 (3.74%)	1 (4%)	3 (3.66%)
“Territory under Ukrainian control, but military present”	N/A	12 (11.20%)	7 (28%)	5 (6.10%)
“Territory under Ukrainian control, but no military”	N/A	38 (35.50%)	15 (60%)	23 (28.00%)
No Response	N/A	53 (49.50%)	2 (8%)	51 (62.20%)
Family in Poland				
Both parents	N/A	16 (14.95%)	11 (44%)	5 (6.10%)
One parent	N/A	22 (20.56%)	12 (48%)	10 (12.20%)
Grandparent(s)	N/A	12 (11.21%)	7 (28%)	5 (6.10%)
Sibling(s)	N/A	17 (15.89%)	10 (40%)	7 (8.54%)
Other	N/A	10 (9.35%)	4 (16%)	6 (7.32%)
No response	N/A	61 (57.00%)	1 (4%)	60 (73.20%)

Among Ukrainian youth who were eligible and consented to research participation (N = 107), about half identified as a girl (58.90%) and were between the ages of 13–15 (53.30%). Among Ukrainian youth recruited in Poland who completed the pre‐SSI questionnaires (N = 25), 76.00% indicated a refugee status, 84.00% reported having arrived in Poland after the Russian military's invasion on February 24th, 2022, 88.00% relocated from territory under Ukrainian control, and youth overall arrived with various family members (see Table 2). Among Ukrainian youth recruited in Ukraine who completed the pre‐SSI questionnaires (N = 82), 50.00% declined refugee status, while 6.10% (*n* = 5) endorsed being refugees, and 43.90% of youth did not respond. About 3.66% *(n* = 3) indicated living in occupied territory and 34.10% reported living in territory under Ukrainian control, while 62.20% did not identify their area of residence in Ukraine. See Table 2 for a full breakdown of all demographic variables.

As determined using an SMFQ cut‐off score of 11 for early adolescents (see Stoep et al., 2005) to distinguish between those in need of support and those who do not need support, Polish youth who completed pre‐SSI questionnaires reported levels of depression right at the cut‐off (M = 11.83, SD = 6.97), on average. Levels of depression among Ukrainian youth in Poland (M = 12.86, SD = 5.14) and Ukraine (M = 9.21, SD = 5.47) were similarly near the cut‐off score of 11. Using a CRIES‐8 cut‐off score of 17 for children and adolescents to differentiate between the potential presence and absence of PTSD‐related symptoms (see Perrin et al., 2005), Polish youth who completed pre‐SSI questionnaires reported elevated levels of trauma exposure (M = 21.57, SD = 9.41), on average. Due to administrative changes during the trial, trauma exposure was only assessed among Ukrainian youth in Poland, of whom levels of trauma exposure were right at the cut‐off (M = 17.67, SD = 7.64), on average. See Table 3 for descriptive statistics of all measures.

**TABLE 3:** Descriptive Statistics of All Measures.

Measure	Polish Youth	All Ukrainian Youth	Ukrainian Youth in Poland	Ukrainian Youth in Ukraine
	*M* (*SD*)	*M* (*SD*)	*M* (*SD*)	*M* (*SD*)
Trauma Exposure	21.57 (9.41)	17.67 (7.64)	17.67 (7.64)	N/A
Depression	11.83 (6.97)	10.21 (5.59)	12.86 (5.14)	9.21 (5.47)
Hopelessness				
Pre‐SSI	4.24 (2.81)	3.07 (2.67)	3.90 (2.52)	2.75 (2.68)
Post‐SSI	3.65 (2.69)	3.46 (3.20)	3.44 (2.75)	3.47 (3.46)
Agency				
Pre‐SSI	16.67 (3.82)	16.85 (5.24)	17.14 (4.60)	16.74 (5.51)
Post‐SSI	17.41 (4.69)	17.06 (5.88)	19.00 (4.67)	15.94 (6.28)
Self‐Hate				
Pre‐SSI	6.27 (4.30)	5.94 (4.55)	6.95 (4.38)	5.52 (4.60)
Post‐SSI	5.72 (4.25)	5.95 (4.66)	6.31 (4.57)	5.74 (4.78)
Perceived Control				
Pre‐SSI	4.02 (3.04)	3.03 (3.19)	3.38 (3.04)	2.87 (3.28)
Post‐SSI	3.07 (2.73)	2.16 (2.77)	2.40 (2.72)	2.03 (2.83)
Positive Mental Health				
Pre‐SSI	16.28 (5.74)	14.86 (5.56)	14.86 (5.56)	N/A
Post‐SSI	16.92 (6.26)	15.29 (4.95)	15.29 (4.95)	N/A
Perceived Change – Hope	3.48 (0.69)	3.68 (1.24)	3.79 (1.03)	3.62 (1.34)
Perceived Change – Problem‐Solving	3.51 (0.71)	3.64 (1.07)	3.68 (0.82)	3.61 (1.18)

*Note*: M is an abbreviation for mean; SD is an abbreviation for standard deviation; N/A is an abbreviation for not applicable.

For youth who accessed Project Personality in Polish (N = 160), n = 154 completed pre‐SSI measures, n = 142 completed the 30‐minute self‐guided SSI, n = 138 answered questions on acceptability, and n = 122 completed post‐SSI measures. Among Polish youth who completed the survey (N = 122), the average number of minutes spent completing the Polish translation of Project Personality was 13.05 (SD = 9.06). Among Polish participants who did not finish the full survey (n = 38), n = 6 discontinued during the pre‐SSI questionnaires, n = 12 discontinued during the SSI, and n = 20 discontinued during the post‐SSI measures.

For youth who accessed Project Personality in Ukrainian (N = 107), n = 98 completed pre‐SSI measures, n = 87 completed the 30‐minute self‐guided SSI, n = 83 answered questions on acceptability, and n = 74 completed post‐SSI measures. Among Ukrainian youth who completed the survey (N = 74), the average number of minutes spent completing the Ukrainian translation of Project Personality was 10.42 (SD = 11.73). Among Ukrainian participants who did not finish the full survey (n = 33), n = 9 discontinued during the pre‐SSI questionnaires, n = 11 discontinued during the SSI, and n = 13 discontinued during the post‐SSI measures.

### Did Youths Perceive YES as Acceptable?

Youths who completed the SSI and the PFS in the Polish translation (n = 138, 86.25%) and the Ukrainian translation (n = 83, 77.57%) found the SSI to be acceptable, both at the individual item level and in overall mean scores. Among both Polish and Ukrainian youth, the SSI was rated as enjoyable (3.84/5.00 and 3.91/5.00, respectively), easy to understand (4.02/5.00 and 4.14/5.00, respectively), easy to use (3.58/5.00 and 3.96/5.00, respectively), likely to help their peers (3.99/5.00 and 3.96/5.00, respectively), and worth recommending to a friend (3.70/5.00 and 3.98/5.00, respectively). Furthermore, both Polish and Ukrainian youth endorsed trying their hardest while completing the SSI (3.60/5.00 and 3.41/5.00, respectively) and agreed with the SSI's message (4.02/5.00 and 4.00/5.00, respectively). See Table 4 for a full breakdown of all acceptability scores, demonstrating how the pre‐registered benchmark for a score of 3.00 out of 5.00 was passed, reflecting perceived SSI acceptability (see https://osf.io/9h84t/).

**TABLE 4:** Means and standard deviations of Program Feedback Scale items among Polish and Ukrainian youth, mean (SD).

Item	Polish Youth	All Ukrainian Youth	Ukrainian Youth in Poland	Ukrainian Youth in Ukraine
Enjoy	3.84 (0.87)	3.91 (1.01)	3.96 (1.11)	3.90 (0.99)
Understood	4.02 (0.91)	4.14 (0.92)	4.22 (0.85)	4.10 (0.95)
Easy to use	3.58 (0.97)	3.96 (1.02)	3.74 (0.92)	4.05 (1.05)
Tried hardest	3.60 (0.97)	3.41 (1.20)	3.43 (1.08)	3.40 (1.26)
Helpful	3.99 (0.89)	3.96 (1.05)	4.00 (1.04)	3.95 (1.07)
Recommend to friend	3.70 (1.02)	3.98 (1.11)	3.96 (1.02)	3.98 (1.15)
Agree with message	4.02 (0.85)	4.00 (0.98)	4.00 (0.91)	4.00 (1.01)
Full scale	3.82 (0.68)	3.91 (0.84)	3.90 (0.69)	3.91 (0.90)

*Note*: SD is an abbreviation for standard deviation; scores reported on a scale from 1 to 5.

### Did Hopelessness, Agency, Self‐Hatred, Perceived Control, and Positive Mental Health Improve from Before to After YES?

To optimize the validity and accuracy of the effect size calculations, participant data were filtered to remove responses demonstrating a “straightlining” pattern across both pre‐ and post‐SSI assessments of each mental health outcome of interest (i.e., hopelessness, agency, self‐hatred, perceived control, positive mental health). Among Polish youth, 7.38% (9/122) of hopelessness data, 3.28% (4/122) of agency data, and 2.46% (3/122) of self‐hate data exhibited straightlining across pre‐ and post‐SSI responses for each measure. Among Ukrainian youth, 20.27% (15/74) of hopelessness data, 13.51% (10/74) of agency data, and 2.70% (2/74) of self‐hate data exhibited straightlining across pre‐ and post‐SSI responses for each measure. Straightlining was not examined in positive mental health data because not all participants received the measure, and perceived control data consisted of a single‐item question, so straightlining could not occur. The results reported subsequently reflect the effect sizes observed after the removal of straightlining responses for each measure.

SSI short‐term utility was tested to detect pre‐ to post‐SSI change by calculating effect sizes (both Cohen's dav and dz) and 95% confidence intervals for each outcome of interest. Youth who completed the Polish translation of the SSI reported significant improvements in agency, self‐hate, perceived control, and positive mental health from before to after completing the SSI. For overall increases in agency, small‐to‐medium effects emerged (2‐tailed t[102] = −3.02, p < .01; 95% CI 0.10, 0.49; Cohen's dz = .30), with post‐SSI agency showing a 21% chance of being higher than pre‐SSI agency, per the “common language effect size” estimate (see Lakens, 2013). For overall reductions in self‐hate, small‐to‐medium effects emerged (t[87] = 3.37, p < .01; 95% CI 0.14, 0.57; Cohen's dz = .36), with post‐SSI self‐hate showing a 17% chance of being lower than pre‐SSI self‐hate. For overall improvements in perceived control, medium effects (t[97] = 5.99, p < .001; 95% CI 0.39, 0.82; Cohen's dz = .61), with post‐SSI reports of “feeling out of control” showing a 31% chance of being lower than pre‐SSI reports of “feeling out of control.” For overall increases in positive mental health, medium effects emerged (t[100] = −5.32, p < .001; 95% CI 0.32, 0.74; Cohen's dz = .53), with post‐SSI positive mental health levels showing a 23% chance of being higher than pre‐SSI positive mental health levels.

Youth who completed the Ukrainian translation of the SSI reported significant improvements in perceived control from before to after completing the SSI. For overall improvements in perceived control, medium effects emerged (t[41] = 3.04, p < .01; 95% CI 0.15, 0.79; Cohen's dz = .47), with post‐SSI reports of “feeling out of control” showing a 29% chance of being lower than pre‐SSI reports of “feeling out of control.” Youth who completed the Ukrainian translation did not report significant changes in any other target outcome. Notably, when examining within‐group differences among Ukrainian youth, youth recruited in Poland did not report any significant changes for any outcomes, including perceived control, though this was likely related to small, insufficiently powered sample sizes (e.g., n = 18 for hopelessness, n = 15 for perceived control, etc.; see Table 5). However, youth recruited in Ukraine reported significant improvements in perceived control from before to after completing the SSI. For overall improvements in perceived control among youth recruited in Ukraine, medium effects emerged (t[26] = 2.23, p < .05; 95% CI 0.03, 0.82; Cohen's dz = .43), with post‐SSI reports of “feeling out of control” showing a 24% chance of being lower than pre‐SSI reports of “feeling out of control.” Youth in Ukraine did not report any other significant changes in target outcomes.

**TABLE 5:** Means, standard deviations, and effect sizes across Polish and Ukrainian youth; mean (SD) or effect size (95% CI).

Outcome	Polish Youth	All Ukrainian Youth	Ukrainian Youth in Poland	Ukrainian Youth in Ukraine
**Hopelessness**	*n* = 103	*n* = 50	*n* = 18	*n* = 32
Baseline	4.24 (2.81)	3.07 (2.67)	3.90 (2.52)	2.75 (2.68)
Post‐SSI	3.65 (2.69)	3.46 (3.20)	3.44 (2.75)	3.47 (3.46)
*d*_av_	.15 (−0.04, 0.34)	.00 (−0.28, 0.28)	.17 (−0.30, 0.63)	.08 (−0.27, 0.43)
*d*_z_	.17 (−0.02, 0.36)	.00 (−0.28, 0.28)	.19 (−0.27, 0.66)	.06 (−0.29, 0.40)
**Agency**	*n* = 103	*n* = 48	*n* = 18	*n* = 30
Baseline	16.67 (3.82)	16.85 (5.24)	17.14 (4.60)	16.74 (5.51)
Post‐SSI	17.41 (4.69)	17.06 (5.88)	19.00 (4.67)	15.94 (6.28)
*d*_av_	.21 (0.02, 0.41)**	.20 (−0.09, 0.48)	.37 (−0.12, 0.84)	.12 (−0.24, 0.47)
*d*_z_	.30 (0.10, 0.49)**	.21 (−0.08, 0.49)	.42 (−0.07, 0.89)	.12 (−0.24, 0.47)
**Self‐Hate**	*n* = 88	*n* = 43	*n* = 16	*n* = 27
Baseline	6.27 (4.30)	5.94 (4.55)	6.95 (4.38)	5.52 (4.60)
Post‐SSI	5.72 (4.25)	5.95 (4.66)	6.31 (4.57)	5.74 (4.78)
*d*_av_	.17 (−0.04, 0.38)**	.12 (−0.18, 0.42)	.33 (−0.18, 0.83)	.01 (−0.37, 0.38)
*d*_z_	.36 (0.14, 0.57)**	.25 (−0.05, 0.55)	.52 (−0.01, 1.03)	.02 (−0.36, 0.40)
**Perceived Control**	*n* = 98	*n* = 42	*n* = 15	*n* = 27
Baseline	4.02 (3.04)	3.03 (3.19)	3.38 (3.04)	2.87 (3.28)
Post‐SSI	3.07 (2.73)	2.15 (2.77)	2.40 (2.72)	2.03 (2.83)
*d*_av_	.31 (0.10, 0.51)***	.29 (−0.02, 0.60)**	.39 (−0.15, 0.90)	.24 (−0.14, 0.62)*
*d*_z_	.61 (0.39, 0.82)***	.47 (0.15, 0.79)**	.52 (−0.03, 1.06)	.43 (0.03, 0.82)*
**Positive MH**	*n* = 101	*n* = 17	*n* = 17	*n* = N/A
Baseline	16.28 (5.74)	14.86 (5.56)	14.86 (5.56)	N/A
Post‐SSI	16.92 (6.26)	15.29 (4.95)	15.29 (4.95)	N/A
*d*_av_	.23 (0.03, 0.43)***	.26 (−0.22, 0.74)	.26 (−0.22, 0.74)	N/A
*d*_z_	.53 (0.32, 0.74)***	.34 (−0.15, 0.83)	.34 (−0.15, 0.83)	N/A

*Note*: SD is an abbreviation for standard deviation; CI is an abbreviation for confidence interval; *d*_z_ and *d*_av_ represent Cohen's *d* effect sizes; MH is an abbreviation for mental health; * indicates significance of *p* < .05; ** indicates significance of *p* < .01; *** indicates significance of *p* < .001.

### Did Youths Subjectively Detect Changes in Hopelessness and Problem‐Solving Ability from Before to After YES?

Among youth who completed the Polish translation of the SSI, after the intervention, 3.42% (5/146) reported feeling “a lot less hopeless,” 37.00% (54/146) felt “a little less hopeless,” 37.70% (55/146) felt “the same amount of hopeless,” 3.42% (5/146) felt “a little more hopeless,” and 0.69% (1/146) felt “much more hopeless” compared with before beginning the SSI. Separately, 5.48% (8/146) of youths reported feeling “a lot more able to solve problems,” 34.20% (50/146) felt “a little more able to solve problems,” 39.70% (58/146) felt “the same amount able to solve problems,” 0.69% (1/146) felt “a little less able to solve problems,” and 1.37% (2/146) felt “much less able to solve problems” compared with before beginning the SSI.

Among youth who completed the Ukrainian translation of the SSI, after the intervention, 19.80% (17/86) reported feeling “a lot less hopeless” (22.70% or 5/22 of those in Poland, 18.80% or 12/64 of those in Ukraine), 20.90% (18/86) felt “a little less hopeless” (36.40% or 8/22 of those in Poland, 15.60% or 10/64 of those in Ukraine), 14.00% (12/86) felt “the same amount of hopeless” (13.60% or 3/22 of those in Poland, 14.10% or 9/64 of those in Ukraine), 4.65% (4/86) felt “a little more hopeless” (13.60% or 3/22 of those in Poland, 1.56% or 1/64 of those in Ukraine), and 5.81% (5/86) felt “much more hopeless” (0% or 0/22 of those in Poland, 7.81% or 5/64 of those in Ukraine) compared with before beginning the SSI. Separately, 16.30% (14/86) of youths reported feeling “a lot more able to solve problems” (13.60% or 3/22 of those in Poland, 17.20% or 11/64 of those in Ukraine), 20.90% (18/86) felt “a little more able to solve problems” (36.40% or 8/22 of those in Poland, 15.60% or 10/64 of those in Ukraine), 23.30% (20/86) felt “the same amount able to solve problems” (31.80% or 7/22 of those in Poland, 20.30% or 13/64 of those in Ukraine), 3.49% (3/86) felt “a little less able to solve problems” (4.55% or 1/22 of those in Poland, 3.12% or 2/64 of those in Ukraine), and 3.49% (3/86) felt “much less able to solve problems” (0% or 0/22 of those in Poland, 4.69% or 3/64 of those in Ukraine) compared with before beginning the SSI.

## DISCUSSION

The current open pilot trial examined the acceptability and short‐term utility of a digital, growth mindset SSI, Project Personality, among Polish and Ukrainian youth, including refugees in Poland and youth remaining in Ukraine. Mental health care across the world is increasingly difficult to access, and findings reviewed here demonstrate the impact and potential of self‐guided SSIs in expanding access to care, especially for youth who need help with their mental health. More importantly, this pilot trial investigated how Project Personality impacted youth currently being affected by the Russo‐Ukrainian war. Differences emerged in how Polish and Ukrainian youth engaged with the SSI, alluding to the importance of accounting for cultural factors when strategically deploying digital, self‐guided SSIs for youth exposed to war, displacement, and sociopolitical stressors. Results from this pilot trial establish directions for future research to further explore how digital, self‐guided SSIs can scale up access to mental health services among war‐impacted youth and communities in neighboring countries, such as with Ukrainian and Polish youth.

Per our *first research question* (i.e., to what extent is the adapted SSI acceptable and feasible for Polish and Ukrainian youth?), most participants who started the SSI completed it (completion rates were 63–80%). Observed completion rates were higher than or comparable to previous naturalistic digital SSI evaluations in other settings (Schleider et al., 2020; Shroff et al., 2023). Additionally, youths rated the SSI as acceptable (enjoyable, understandable, and user‐friendly). Together, high completion and acceptability ratings align with past research suggesting the utility of digital SSIs outside of North America, including those tested in the present study (Buchlmayer et al., 2024; Schleider et al., 2025). Here, high acceptability ratings are especially notable given the compressed timeline under which cultural adaptations were formulated and deployed; likewise, high completion rates are promising given the absence of monetary incentives for participants, which have been shown to substantially increase completion rates in digital SSI evaluations (Cohen & Schleider, 2022). The SSI dissemination strategy used in this trial—namely, allotting class time in schools for youth to voluntarily complete an SSI—likely contributed to relatively high completion rates. School‐based SSI deployment may be a promising longer‐term deployment strategy to streamline SSI uptake among Polish and Ukrainian students.

Results linked to our *second research question* (i.e., is completion of the SSI associated with short‐term improvements in key mental health outcomes?) differed across groups and must be interpreted with contextual considerations. Modest, statistically significant improvements were observed for Polish youth across measures of agency, self‐hate, perceived control, and the positive mental health scale from before to immediately after completing Project Personality. These findings paralleled a previous study on Project Personality conducted with Arabic‐speaking adolescents in Lebanon, predominantly Syrian refugees, that also found moderate effect sizes and significant reductions in hopelessness (d = 0.48) and self‐hate (d = 0.57), and improvements in perceived control (d = 0.50) from pre‐ to post‐SSI (Buchlmayer et al., 2024), demonstrating how digital mental health interventions benefit Arabic‐speaking youth (Raknes et al., 2024). Results reviewed here extend the evidence base on how single‐session programs focusing on growth mindset principles foster small to moderate improvements in mental health (Schleider & Weisz, 2018), including in cultural contexts outside of North America (e.g., Spain: Calvete et al., 2023; Lebanon: Buchlmayer et al., 2024).

However, only perceived control significantly improved for Ukrainian youth, particularly for those in Ukraine, and subgroup differences between Ukrainian youth in Poland versus Ukraine must be interpreted cautiously due to the small and unequal sample sizes (i.e., *n* = 25 and *n* = 82, respectively). While interpreting these results, we considered how the COVID‐19 pandemic and the ongoing war in Ukraine intensified mental health problems among youth, including depression, anxiety, and loneliness, especially among older youth and girls (Domagała‐Zyśk, 2020; Gambin et al., 2023; Korzycka et al., 2021; Wójtowicz‐Szefler et al., 2023). Notably, a 2019 Polish education reform introduced an intensive schooling system, which received some negative student feedback (Karwowski & Milerski, 2021a, 2021b) like remarks about unrealistic academic expectations, and combined with economic stressors to exacerbate emotional exhaustion (Gaspar de Matos et al., 2021). Ukrainian youth faced compounded stressors as they integrated into Polish schools, were challenged by language barriers, and exhibited a need for psychological support (Grzelak & Żyro, 2023). Nevertheless, the likelihood of greater severity of mental health problems among Ukrainian youth does not guarantee that SSI benefits will be reduced, due to prior research documenting how more severe difficulties at baseline do not necessarily indicate SSI response will be decreased, relative to those with less severe difficulties prior to starting the SSI (Schleider et al., 2025).

The observed differences in intervention outcomes between Polish and Ukrainian youth may be partly explained by the psychological and sociopolitical contexts each group faces. Ukrainian youth, particularly those displaced and exposed to ongoing war trauma, experience heightened emotional distress and cognitive burden (Williamson & Murphy, 2025), which may limit their immediate responsiveness to brief interventions. A recent review by Carpiniello (2023) examined the psychological impact of war and armed conflicts, focusing on recent evidence. The study highlights that exposure to armed conflict significantly increases the risk of mental health issues like anxiety, depression, and PTSD, with women and children often being the most vulnerable. Moreover, the review also emphasizes the *long‐term effects* of war‐related stressors on mental health and the need for ongoing mental health support. Similar findings have been found by Amsalem et al. (2025), who found that individuals exposed to armed conflict experience significantly higher rates of anxiety, depression, and PTSD compared to those not exposed. Refugee youth in Poland face additional stressors such as language barriers and social integration challenges, possibly compounding these effects. In contrast, Polish youth, while affected by regional instability, may have greater psychological stability and fewer direct trauma exposures, facilitating broader benefits from the intervention. Moreover, the timing of trauma exposure (i.e., ongoing for Ukrainian youth in Ukraine versus more post‐migration for Ukrainian youth in Poland or indirect for Polish youth) likely influences receptivity to the intervention, as ongoing trauma has been shown to reduce cognitive and emotional resources necessary for therapeutic engagement, by disrupting adaptive functioning, which may reduce the effectiveness of brief psychological interventions among youth in active conflict zones (Masten & Narayan, 2012). In this respect, Bürgin et al. (2022) emphasized that the infliction of war and military aggression has a persistent impact on the physical and mental health and well‐being of youth, with long‐term consequences, therefore highlighting the necessity of “multilevel, need‐oriented, and trauma‐informed approaches to regaining and sustaining their outer and inner security after exposure to the trauma of war” (Bürgin et al., 2022, p. 845).

Regarding our *third research question* (i.e., how do the unique sociocultural and war‐related circumstances of Polish and Ukrainian youth, particularly refugees, influence their engagement with and outcomes from a brief digital psychotherapeutic intervention?), we found comparable rates of traumatic exposure between Polish and Ukrainian youth, and similarly, participants reported small‐to‐medium benefits on average, irrespective of trauma exposure per the CRIES‐8. Though Polish youth exhibited significant improvements across multiple outcomes and Ukrainian youth only exhibited significant improvements in perceived control, the lack of significant change in outcomes among Ukrainian youth may be attributed to insufficient, small sample sizes. Nonetheless, variance in effect sizes across Ukrainian subgroups alludes to the heterogeneity of experiences among Ukrainians. Furthermore, due to the administrative complications that limited our use of the CRIES‐8 with all participants, we cannot make definitive claims about the influence of trauma, war, or displacement on intervention effectiveness. Future research replicating this study should consider conducting moderation analyses to investigate if trauma exposure moderates the SSI's short‐term improvements. The present study's findings align with prior research affirming that youth with varying levels of symptom severity and comorbid mental health difficulties can still benefit from digital, self‐guided SSIs. For instance, in past studies we have found that youth with and without suicidal ideation and behaviors found the SSI acceptable and effective in reducing distress and suicidality (Schleider et al., 2020). Importantly, the ability of SSIs to overcome common barriers to care while delivering support in a non‐stigmatizing context (e.g., self‐guided, private, anonymous) can create approachable, safe environments for youth to engage with therapeutic content and build coping skills. This might be particularly useful for populations exposed to traumatic stressors, such as Polish and Ukrainian youth facing displacement, proximity to conflict zones, and fear for relatives near active frontlines, which may limit the impact of brief mental health supports (Masten & Narayan, 2012). Notably, during focus group interviews, youth remaining in Ukraine reported fears related to the threat of war, whereas Ukrainian youth in Poland described challenges with assimilation and language barriers (Sangalang et al., 2019). More research is needed to investigate if specific traumatic stressors have differential effects on SSI effectiveness.

Importantly, without a control or comparison group and without randomization to intervention conditions, the interpretation of intervention efficacy must account for the possibility of non‐specific effects. Differences in the SSI's effects on target outcomes may have resulted from the passage of time since a youth's exposure to traumatic stressors or from potentially calming effects of completing a mental health intervention in a controlled environment (i.e., in a classroom, with peers, under the supervision of mental health researchers). Without a comparison condition, we cannot conclude whether any observed improvements occurred from the SSI's effectiveness, from positive reactions to completing a digital SSI, or from a desire to ‘perform well’ because of researcher presence (i.e., the Hawthorne effect; Berkhout et al., 2022). This pilot trial established a foundation for future randomized controlled trials (RCTs) and longitudinal follow‐ups to evaluate the SSI's potential for improving target outcomes immediately after completion and sustaining improvements over time.

### Limitations

Insights gained from these results must be interpreted in the context of various study limitations. First, the study employed a relatively small and opportunistically recruited sample in both countries, which limits the generalizability of our findings. Although our sample sizes were sufficient for preliminary analyses, the numbers were not large enough to detect more subtle effects or differences between subgroups, especially between Ukrainian youth in Poland versus those remaining in Ukraine. Thus, the geographical location in certain regions of the country might reveal further discrepancies. This was particularly evident when comparing psychological outcomes; some changes may have occurred, but our small sample size did not have enough statistical power to detect them reliably.

Second, we did not collect detailed background information about participants beyond basic demographics like age, gender, nationality, and refugee status. That means some important variables—including participants' emotional regulation skills, social competencies or difficulties, history of mental health challenges, academic stressors, or prior use of psychological or pedagogical support services—were not assessed. These factors could have impacted both engagement with the intervention and mental health outcomes. Furthermore, although the intervention was received well and completion rates were relatively high, we did not employ a control group nor a randomized design. As a result, causal interpretations regarding the effectiveness of the intervention cannot be made.

A final limitation worth noting was the interrupted administration of the CRIES‐8, which limited our ability to thoroughly investigate our third research question on the influence of the Russo‐Ukrainian war and its sociocultural implications. Without assessing trauma exposure for all participants across Polish and Ukrainian samples, we could not definitively determine if trauma moderated the short‐term improvements associated with completing Project Personality. Among Polish youth (*n* = 160), the CRIES‐8 was completed by 134 Polish participants; among Ukrainian youth in Poland (*n* = 25), the CRIES‐8 was completed by 21 Ukrainian participants; and no Ukrainian youth in Ukraine (*n* = 82) completed the CRIES‐8. Completion rates and acceptability ratings of Project Personality were high, suggesting that war‐related circumstances may not have inhibited engagement with the digital, self‐guided SSI. Yet, further research is needed to statistically determine if baseline levels of exposure to traumatic stress correlate with differential outcomes. Understanding how traumatic stressors, such as those related to active conflict, displacement, and economic strain, impact intervention effects is imperative to the design and dissemination of brief mental health supports for adolescents experiencing war‐related crises. Awareness of the specific effects of conflict and violence on SSI effectiveness can inform both intervention tailoring and implementation efforts to strategically enhance intervention uptake and engagement. Future studies should assess trauma histories from all participants involved, as well as assess for any trauma‐related symptoms from all participants, to determine both how trauma exposure might moderate Project Personality's effects on improving target outcomes, and if the SSI's effects reduce trauma‐related symptoms.

### Future Directions

Going forward, it would be valuable to repeat this kind of intervention in larger, more diverse samples, and to include measures that capture participants' emotional profiles and mental health history. Ideally, future studies could also follow up after a few weeks or months to see whether any changes are sustained over time. Including a control group could clarify how potentially confounding variables contribute to the observed improvements, and a randomized controlled trial would allow us to establish causal claims about the effects of this growth mindset SSI on Polish and Ukrainian youth mental health. Results from this pilot trial can guide future efforts to examine the benefits of digital SSIs for Polish and Ukrainian youth, as well as for youth experiencing active conflict, like the Russo‐Ukrainian war.

Additionally, given some concerns over excessive internet use and exposure to cyberbullying or violent content online (Grzelak & Żyro, 2023; Szaban, 2023), future research should capitalize on the brevity of self‐guided SSIs to deliver care in shorter time periods, limiting youths' time spent online. Internet‐based interventions hold potential for population‐level scalability that expands access to prevention and early intervention globally, and further research is needed to explore how the benefits gleaned from digital SSIs can be maximized while minimizing overuse of the internet. Considering how loneliness has been identified as a key factor in deteriorating mental health among Polish populations (Dziedzic et al., 2021; Kobos et al., 2022), future efforts to implement digital SSIs should explore how group settings and school‐based delivery can increase community building and engagement in interpersonal relationships.

## CONCLUSION

The findings from this open pilot trial suggest that the growth mindset‐based SSI, Project Personality, is an acceptable tool for improving mental health outcomes among Polish and Ukrainian youth. Despite the challenges faced by youth in these countries, including the direct impacts of war and displacement, the intervention was generally well‐received, with high completion rates and positive feedback on its accessibility, usability, and perceived value. These outcomes align with previous research, within the United States and internationally (i.e., among Syrian refugees and Lebanese youth), demonstrating the potential of SSIs in addressing mental health challenges across diverse cultural and political contexts.

This study outlines a protocol for conducting rapid cultural adaptations of a brief intervention in the context of a crisis—here, the Russo‐Ukrainian war—to rapidly disseminate evidence‐based mental health support to adolescents experiencing displacement, conflict‐related uncertainty, and mental health problems. The use of focus group interviews with both professionals as well as adolescents served to engage in community‐focused methods for partnering with the target population meant to benefit from the intervention. After collecting feedback and implementing the cultural adaptations, the self‐guided, digital SSI was made immediately available online and for free, to help overcome common barriers to mental health care (e.g., provider shortages, inability to afford care, lack of transportation to in‐person services). Polish youth who participated in the SSI showed notable improvements in several key mental health indicators, including reductions in self‐hate and increases in agency, perceived control, and positive mental health. These improvements align with the intervention's intended goals, which were rooted in fostering a growth mindset and reframing adversity as an opportunity for personal growth. The moderate effect sizes observed in this group support the potential of SSIs to achieve meaningful psychological change in a brief and scalable format. In contrast, the impact of the intervention was more varied among Ukrainian youth, particularly among those who had been displaced. While significant improvements in perceived control were noted, other mental health indicators, such as hopelessness, self‐hate, and agency, did not show the same level of change, possibly being under the influence of the complex interplay between war trauma, displacement, and mental health. Youth who had fled Ukraine and were resettling in Poland reported higher levels of distress, possibly due to the compounded challenges of adjusting to a new country and educational system, while youth who remained in Ukraine reported concerns focused on the proximity of active conflict zones. However, definitive claims could not be made about the differences between Ukrainian youth in Poland versus Ukraine, due to insufficient, small sample sizes.

Positive reception of Project Personality, as evidenced by high acceptability ratings and completion rates, suggests that youth in or near active conflict zones and those endorsing refugee status may reap meaningful benefits after completing a single‐session activity designed to boost their mental health. Youth facing conflict‐related hardships (e.g., displacement, concerns for survival of their family, etc.) may encounter unique traumatic stressors that have the potential to influence engagement with and effectiveness of a digital SSI. The study reviewed here outlined one approach for rapidly adapting an existing evidence‐based support program to enhance agency, perceptions of change, and positive mental health while decreasing hopelessness, self‐hatred, and depression. Further attention is needed to better understand the specific sociocultural dynamics of displacement, economic strain, and active conflict that contribute to the significant increases in demands for mental health care among adolescents, particularly those in Central and Eastern Europe. Future work should build on these results to continue ensuring the needs of youth are met when needed, and in ways that center agency and autonomy.

## CONFLICT OF INTEREST STATEMENT

JLS serves on the Scientific Advisory Board for Walden Wise and the Clinical Advisory Board for Koko; receives consulting fees from Kooth, LLC and Woebot Health; and receives book royalties from New Harbinger, Oxford University Press, and Little Brown Book Group; serves as Mindly co‐founder and chief scientific advisor. No Mindly products are discussed, referenced, or used in the present research. There are no other conflicts of interest to report for this manuscript.

## ETHICS STATEMENT

This manuscript adheres to all ethical principles outlined by the American Psychological Association (*Ethical principles of psychologists and code of conduct*, APA, 2017).

## Data Availability

The data that support the findings of this study are openly available in the Open Science Framework at https://doi.org/10.17605/OSF.IO/9H84T (or use https://osf.io/9h84t/).
